# Case report and literature review: Small bowel intussusception due to solitary metachronous metastasis from renal cell carcinoma

**DOI:** 10.3389/fonc.2022.1072485

**Published:** 2022-12-19

**Authors:** Wenming Yang, Zhaolun Cai, Pan Nie, Tao Yuan, Hang Zhou, Qiang Du, Siyuan Qiu, Jianhao Zhang, Lie Yang

**Affiliations:** ^1^ Division of Gastrointestinal Surgery, Department of General Surgery, West China Hospital, Sichuan University, Chengdu, China; ^2^ Gastric Cancer Center, Department of General Surgery, West China Hospital, Sichuan University, Chengdu, China; ^3^ Department of Gastrointestinal Surgery, The Third People’s Hospital of Chengdu, Chengdu, China; ^4^ Department of Anesthesiology, West China Hospital, Sichuan University, Chengdu, China

**Keywords:** surgical oncology, case report, renal cell carcinoma, solitary metastasis, adult intussusception

## Abstract

**Introduction:**

Solitary metachronous small bowel metastasis from renal cell carcinoma (RCC) is rare. In contrast to idiopathic intussusception frequently occurring in children, adult intussusception is fairly uncommon and usually indicates a malignancy.

**Case presentation:**

We presented an 84-year-old man with small bowel intussusception and obstruction due to a solitary metachronous metastasis from RCC. Computed tomography with intravenous contrast revealed small bowel obstruction and a 4 × 4 cm intraluminal soft-tissue mass with moderate enhancement. During urgent exploratory laparotomy, a pedunculated tumor of the distal ileum was found to be the lead point of intussusception. Hence, reduction of the intestinal invagination and segmental resection of the ileum with functional end-to-end anastomosis were performed. Histological examination finally confirmed the diagnosis. The postoperative recovery was uneventful. The patient was discharged without any complications on postoperative day 6.

**Conclusion:**

The case report highlights the rarity of solitary metachronous small bowel metastases from RCC and suggests that life-long follow-up of RCC patients is critical due to its unpredictable behavior and the possibility of a long period of dormancy. Complete surgical resection remains the mainstay treatment for such patients.

## Introduction

Renal cell carcinoma (RCC) is the third most frequently diagnosed cancer among urological tumors, with an estimated annual incidence of 0.4 million cases worldwide (1). RCC is a male-predominant (2:1 ratio) malignancy. It appears between 60 and 80 years old, with a median age of approximately 64 years and a near-normal distribution (2). Clear cell RCC (ccRCC) represents the most prevalent histological subtype of RCC (70-80%) and accounts for most RCC-related deaths (3). Partial or radical nephrectomy remains the gold standard in localized ccRCC therapy (4).

Nearly one-third of localized ccRCC patients will eventually develop metastases even after curative nephrectomy (5). Distant metastases in metastatic RCC (mRCC) patients are most frequently found in the lung (45.2%), followed by the bone (29.5%), lymph node (21.8%), liver (20.3%), and adrenal gland (8.9%), whereas the proportion of small bowel metastasis is extremely low (1.1%) (6). Portocaval venous shunts may facilitate small bowel involvement. Metachronous metastasis of RCC may occur more than a decade after the initial presentation and diagnosis. Despite significant progress in systemic treatment over the past two decades, *en bloc* resection of the oligometastatic disease is still a curative option for late RCC metastasis (≥ 2 years) (7).

Adults have intussusception less frequently than children, accounting for less than 5% of all intussusception and 1% of bowel obstruction cases (8). The most frequent pathologic condition that causes adult intussusception is malignancy, and the small bowel is the most affected site (9, 10). Nevertheless, gastrointestinal tract metastasis usually develops from breast cancer, melanoma, lung cancer, and esophageal squamous cell carcinoma, with the small bowel being the least involved site (2%) (11, 12). As a result, initial reduction combined with surgical resection remains the mainstay treatment in adult small bowel intussusception (13).

Following the principles of the CAse REport (CARE) guidelines (14), we reported a small bowel intussusception ascribed to an intramural cauliflower-like mass that proved to be a metastasis from a ccRCC 5 years after radical nephrectomy. In contrast to our case, most adult small bowel intussusceptions are benign illnesses, while large bowel involvement is likely to be malignant. The present case highlights the need for clinicians to maintain a high index of suspicion of metastasis when assessing the occurrence of new symptoms in these patients with a distant history of presumably curative cancer treatment.

## Case presentation

In September 2021, an 83-year-old male patient presented to the emergency department with intermittent abdominal pain and distention accompanied by cessation of passage of flatus and stool for 3 days. The concurrent medical problems included type 2 diabetes mellitus and pulmonary emphysema for more than 30 years. More significantly, he underwent left radical nephrectomy for ccRCC (pT3aN0M0, Fuhrman Grade 3) 5 years previously. The scheduled four cycles of adjuvant therapy with tyrosine kinase inhibitor sunitinib were discontinued because of patient intolerance. Computed tomography (CT) and serum tumor marker levels showed favorable local control and no signs of distant progression during intensive surveillance. The last follow-up visit was just 6 months before this admission.

**Figure 1:**
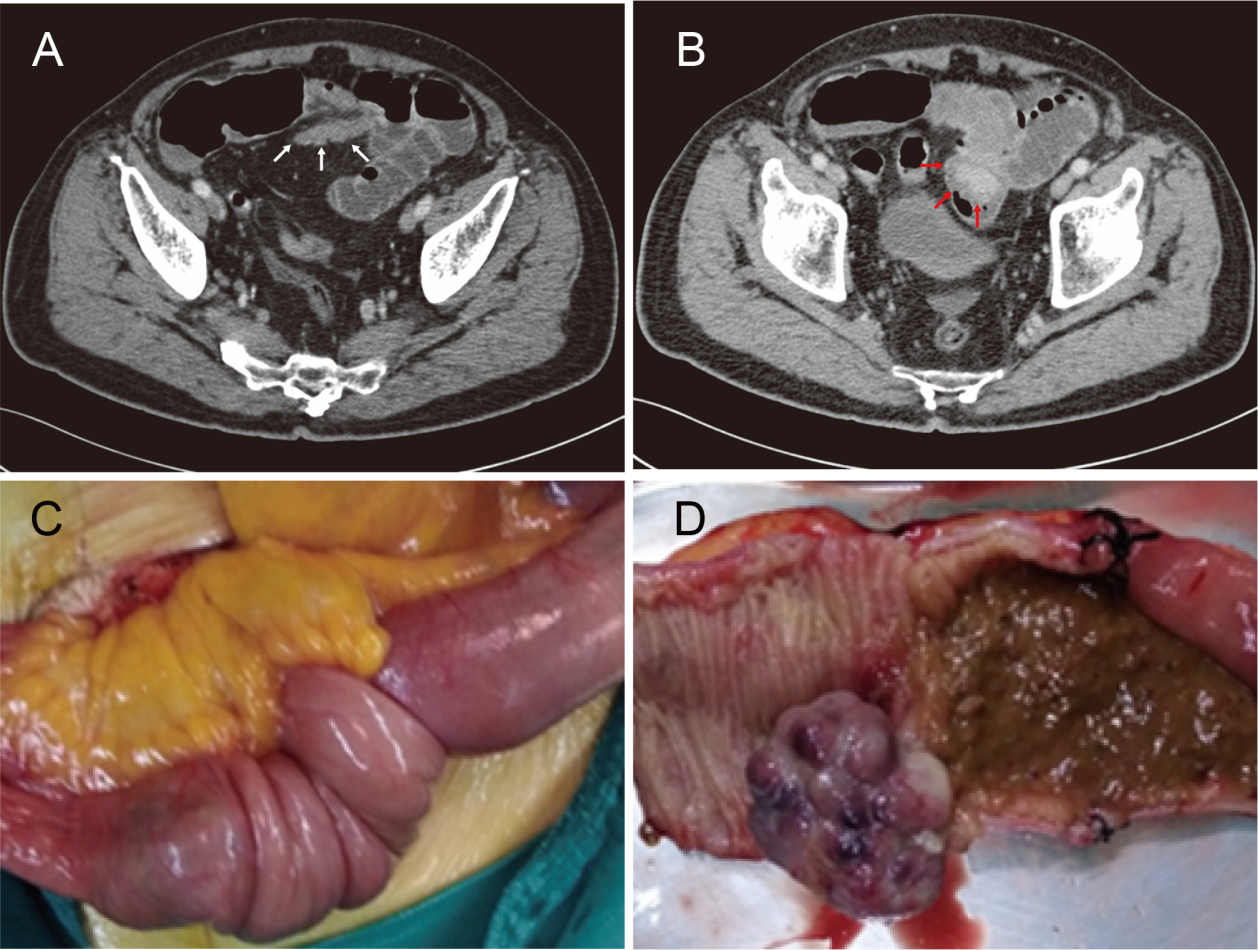
**(A, B)** Computed tomography images demonstrating small bowel obstruction due to intussusception of the distal ileum (*white arrows*) containing an approximately 4 × 4 cm intraluminal soft-tissue mass (*red arrows*). **(C)** An intraoperative photo confirming intussusception whose lead point was a solitary ileal neoplasm. **(D)** Gross image of the surgical specimen with a 4 × 3 × 3-cm taupe cauliflower-like, pedunculated tumor.

On admission, he was afebrile with negative result of coronavirus disease 2019 testing. The blood pressure was 123/84 mmHg, and the heart rate was 105 beats per minute. Physical examination revealed pallor, mild dehydration, abdominal distention, and metallic bowel sounds, without rebound tenderness and palpable abdominal mass. Blood tests indicated iron deficiency anemia (hemoglobin, 9.8 g/dL; reference range, 13.0 – 17.5 g/dL) and an elevated percentage of neutrophils (87%; reference range, 40 – 75%). CT of the chest and abdomen with intravenous contrast demonstrated small bowel obstruction due to intussusception of the distal ileum containing an approximately 4 × 4 cm intraluminal soft-tissue mass with moderate enhancement (**Figures 1A, B**). No lung, bone, or liver metastasis or local relapse of RCC was visible on radiologic examination.

Complying with the multidisciplinary team’s decision, the patient underwent an urgent exploratory laparotomy. After the reduction of intestinal invagination, a solitary ileal neoplasm was found to be the lead point of the intussusception (**Figure 1C**). Hence, segmental resection of the ileum was performed together with primary functional end-to-end anastomosis. Gross examination of the surgical specimen indicated a 4 × 3 × 3-cm taupe cauliflower-like, pedunculated tumor protruding into the ileum (**Figure 1D**).

**Figure 2:**
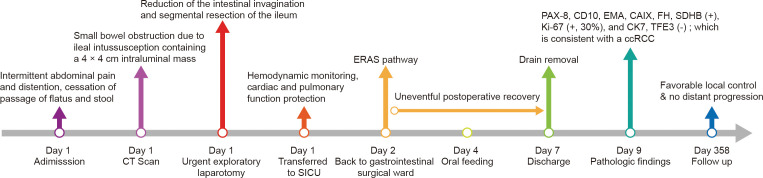
Timeline of the case presentation with relevant data. CT, computed tomography; SICU, surgical intensive care unit; ERAS, enhanced recovery after surgery; ccRCC, clear cell renal cell carcinoma.

Postoperatively, the patient was transferred back to the gastrointestinal surgical ward after spending 1 day in the surgical intensive care unit. Under the guidance of the enhanced recovery after surgery protocol, the patient’s convalescence was unremarkable, with oral feeding beginning on postoperative day (POD) 3 and discharge on POD 6. The patient received no additional treatment due to his venerable age and underlying diseases. To date, the 1-year follow-up has been uneventful. The timeline with relevant data from the episode of care is shown in **Figure 2**.

**Figure 3:**
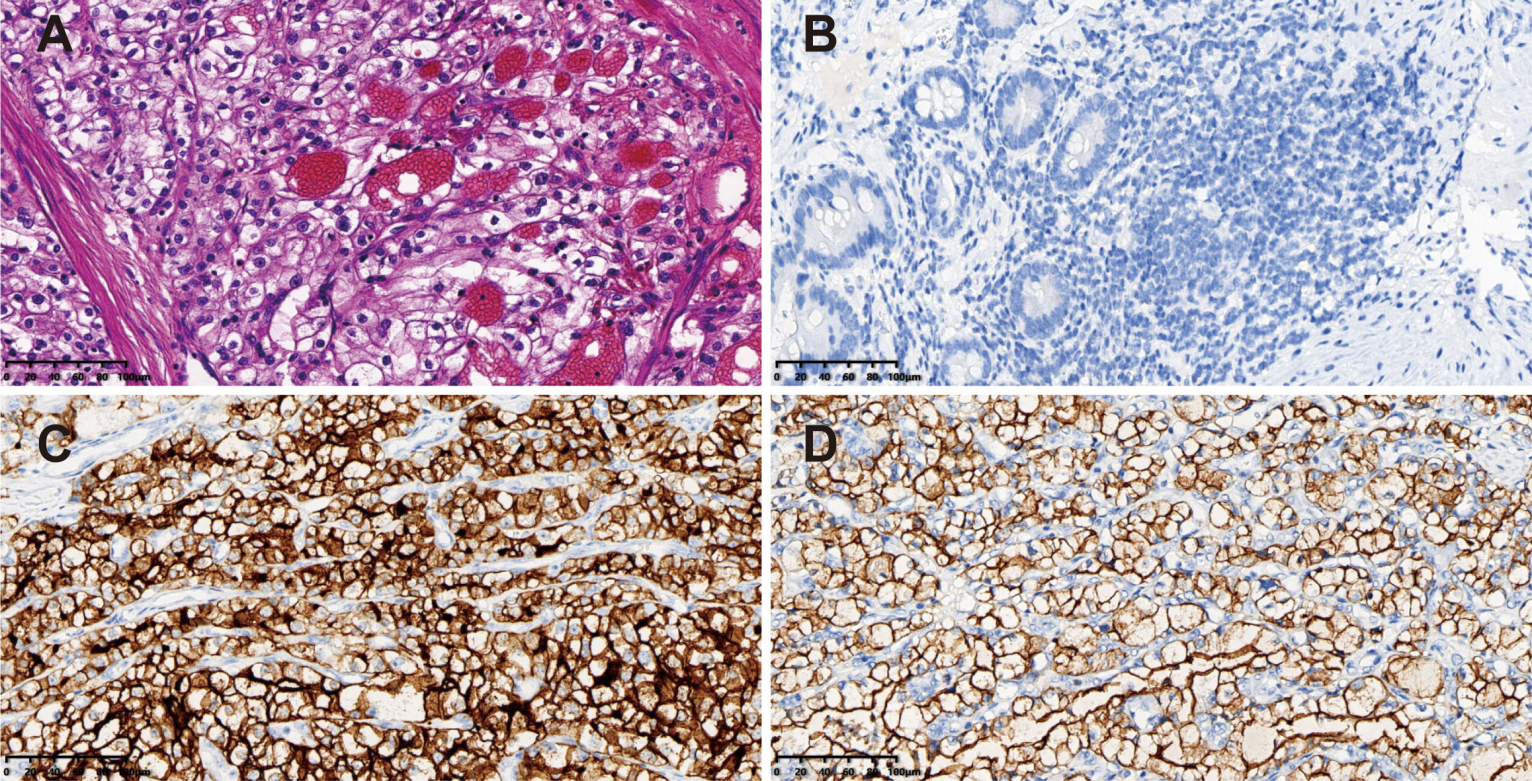
Pathological findings of the surgical specimen. **(A)** Hematoxylin & eosin staining showing compact nests of tumor cells with clear cytoplasm separated by delicate vasculature (magnification power: 20×). Immunohistochemical staining of the tumor revealing **(B)** PAX-8 (+), **(C)** CD10 (+), and **(D)** EMA (+) (magnification power: 20×).

Hematoxylin and eosin staining showed compact nests of tumor cells with clear cytoplasm separated by delicate vasculature (**Figure 3A**). The diagnosis of the ileal metastasis from ccRCC was established *via* the following immunohistochemical results: PAX-8 (+), CD10 (+), EMA (+), CK7 (-), CAIX (+), FH (+), SDHB (+), TFE3 (-), and Ki-67 (+, 30%) (**Figures 3B-D**). A total of 9 mesenteric lymph nodes were identified without tumor involvement. Additionally, genetic testing was declined by the patient’s family members.

## Discussion

A systematic electronic literature search was conducted in the PubMed, Embase (OVID interface), and Cochrane Central Register of Controlled Trials databases using medical subject headings and text words related to “solitary metachronous small bowel metastasis from RCC” to obtain relevant case reports published through 10 May 2022. The term “solitary metachronous small bowel metastasis from RCC” was defined as the small bowel metastasis diagnosed more than 6 months after partial or radical nephrectomy for localized RCC. The search strategy and syntax for the PubMed database are shown in the Supplementary material. Papers with non-English language, animal subjects, or unavailable full text were excluded. The general characteristics of the included studies were extracted and entered into a preplanned electronic form (**Table 1**). The pooled data were interpreted in a descriptive and narrative manner.

**Table 1:** General characteristics of the reported cases of solitary metachronous small bowel metastasis from RCC.

First author& Reference #	Year	Country	Age (years)& Gender	Years post-nephrectomy	Location of metastasis	Clinical presentation	Diagnostic tools	Type of RCC	Treatment Strategy	Follow-up period
Starr A (15)	1952	USA	72 F	20	jejunum	anemia, occult bleeding in the stool	SBS & surgical exploration	clear cell	surgery	/
Nyhan AL (16)	1987	USA	17 M	1	duodenum	massive upper gastrointestinal bleeding	arteriography & endoscopy	/	hemostasis	6 months
Robertson GS (17)	1990	USA	70 M	12	duodenum	weakness, melena	endoscopy & CT	/	surgery	/
Toh SK (18)	1996	UK	69 F	10	duodenum	lethargy, colicky abdominal pain, indigestion, anorexia, weight loss, anemia	surgical exploration & pathologic examination	clear cell	surgery	6 months
Leslie KA (19)	1996	USA	78 F	10	duodenum	upper abdominal discomfort, pruritus	ERCP	clear cell	surgery	2.5 years
Janzen RM (20)	1998	Canada	75 M	17.5	duodenum	maroon-colored stools, anemia	ERCP with side-viewing endoscope	clear cell	surgery	/
Nguyen BD (21)	1998	USA	67 M	20	ileum	intestinal bleeding	scintigraphy	/	surgery	/
Masselli G (22)	2004	Italy	75 F	4	ileum	nausea, melena, loose stools	MRE	/	surgery	/
Chang WT (23)	2004	China	63 F	9	duodenum	upper gastrointestinal bleeding	pathologic examination of frozen sections	clear cell	surgery	10 moths
Bahli ZM (24)	2007	UK	65 F	1	jejunum	tiredness, weight loss, intermittent abdominal pain	CE	/	surgery	/
Adamo R (25)	2008	USA	86 F	13	duodenum	fatigue, anemia, anorexia, early satiety, weight loss	endoscopy & biopsy	clear cell	surgery	7 moths
Takeda T (26)	2011	Japan	75 M	6	jejunum	tarry stools	CE & DBE	clear cell	surgery	6 months
Karahan N (27)	2011	Turkey	69 M	6	jejunum	bloody vomiting	endoscopy & biopsy	/	surgery	/
Vazquez C (28)	2011	Uruguay	68 M	1	jejunum	gastrointestinal bleeding	CE	clear cell	surgery	/
Cohen DL (29)	2013	USA	elderly M	9	jejunum	iron deficiency anemia, intermittent rectal bleeding	CE	clear cell	surgery	/
Chowdhury SD (30)	2014	India	middle-aged M	6	duodenum	cholestatic jaundice, recurrent cholangitis	endoscopy & biopsy	clear cell	PTBD	/
Ismail I (31)	2015	Australia	66 M	19	jejunum	vomiting, abdominal pain	SBS & pathologic examination	clear cell	surgery	18 months
Segura UV (32)	2017	Mexico	48 F	1	duodenum	burning, sharp epigastric pain, hematemesis, melaena	endoscopy & biopsy	clear cell	discharge**#**	1 week**※**
Boullosa PE (33)	2017	Spain	71 F	6	jejunum	melena, anemia	CE & CT	clear cell	surgery	13 months
Mundath V (34)	2017	India	68 F	4	jejunum	abdominal pain	CT & surgical exploration	clear cell	surgery	6 months**※**
Ignatavicius P (35)	2018	Lithuania	62 M	0.66	duodenum	upper abdominal pain jaundice, general weakness, gastric outlet obstruction	duodenoscopy & biopsy	clear cell	surgery	14 years
Lin KH (36)	2020	China	59 M	3	jejunum	intermittent tarry stool	CE	clear cell	surgery	/

**#**The patient requested voluntary discharge from the hospital. **※**The patient died.

RCC, renal cell carcinoma; SBS, small bowel series; CT, computed tomography; ERCP, endoplasmic retrograde cholangiopancreatography; MRE, magnetic resonance enteroclysis; CE, capsule endoscopy; DBE, double-balloon enteroscopy; PTBD, percutaneous transhepatic biliary drainage.

Finally, 48 potential studies were included for the evaluation, in which 21 cases with solitary metachronous small bowel metastases from RCC that had been reported between 1952 and 2020 met the inclusion criteria (15–34, 36, 37). The other cases in different publications were excluded due to synchronous, multiple metastases, or lack of additional radiological examination to check for metastases in other sites. The number of published case reports has increased in the last decade. Patients aged 60 years or older accounted for 81.8% of the enrolled instances (18/22). Like the primary RCCs, clear cell disease was the predominant histological subtype in these small bowel metastases (3). The disease course ranged from 8 months to 20 years post curative nephrectomy. Given the longer survival of RCC patients in the era of targeted therapy and immunotherapy, we are convinced that more late metastases will be identified (38, 39).

Ileal metastases from RCC were rather uncommon in our literature review when compared to duodenal and jejunal metastases. Although some subtypes might exhibit regional lymph node involvement, RCC is notable for hematogenous metastasis (40). Viadana et al. found that RCC first metastasizes to the lung *via* the renal vein and inferior vena cava before spreading systemically to multiple organs (41). However, our case revealed that small bowel metastasis may happen before lung metastasis. Furthermore, a clear cell sarcoma-like tumor of the gastrointestinal tract that primarily affects young-aged to middle-aged individuals is increasingly recognized as a primary neuroectodermal malignancy with clear cell morphology and a predilection for the small bowel (42). Besides, S-100 (+), SOX (+), and *EWSR1* gene fusions are essential to confirming the diagnosis (43).

Small bowel metastases from RCC exhibit various biological characteristics (44). We found that the most typical clinical presentations were gastrointestinal bleeding, melena, and secondary iron deficiency anemia. Willis et al. discovered that metastatic small bowel lesions typically form in the submucosa and cause mucosal rupture and ulceration (Borrmann classification type II or III) and bleeding (11, 45). Some individuals might experience non-specific symptoms, such as intermittent blunt abdominal pain, weakness, and weight loss. In addition, intussusception and perforation could occur in rare cases. Intestinal intussusception is primarily caused by the pedunculated small bowel tumor that originates from the submucosa and represents dimple formation on the serosa. Unlike idiopathic intussusception in children, intussusception in adults and the elderly is uncommon but typically associated with an underlying pathology (37).

The significance of radiological screening should be emphasized in patients with atypical symptoms and an RCC history. Small bowel series is inferior to CT as a practical and effective tool in assessing suspicious small bowel lesions, especially in the emergency setting (34). Capsule endoscopy can facilitate the localization of occult bleeding and intraluminal masses and shorten the diagnosis time (36). Besides, single- or double-balloon enteroscopy can assist in hemostasis and biopsy (26). The Tc-99m tagged red blood cell scan can be used for gastrointestinal hemorrhage to show the hypervascular characteristics of the intestinal lesion and guide additional angiography (21). Exploratory laparoscopy and laparotomy can be performed to help verify the diagnosis after getting informed consent.

Systemic treatment should be considered for individuals with medically inoperable RCC (3). Several immune checkpoint inhibitors were initially tested for RCC in the adjuvant setting (46, 47). Nevertheless, more prospective phase III clinical trials are needed to provide high-quality evidence. It is necessary to conduct clinical and translational studies to determine the phenotypic predictors of response and resistance to each drug. Genetic testing can help select the optimal treatment regimen. Therefore, systemic and surgical treatments must be integrated to achieve a complete response and to obtain a thorough understanding of the biological features of RCC to explore new targets (48).

Meanwhile, complete metastasectomy of synchronous or metachronous solitary RCC metastasis could improve the patient survival (49). Thus, a conventional segmental resection of the ileum was performed with negative margins and lymph nodes in our case. Only one resected specimen reported by Mundath et al. in our literature review revealed the involvement of lymph nodes (34). Endoscopic hemostasis and transcatheter embolization can be utilized as a bridge to surgery in patients with massive gastrointestinal hemorrhage secondary to mRCC (16). Besides, diverting ostomy and gastrojejunostomy are recommended to ease obstruction-related symptoms in unresectable tumors. Definitive radiotherapy rather than no alternative treatment should be considered for RCC patients with medically inoperable duodenal metastases from RCC.

In conclusion, our case suggests that life-long follow-up of RCC patients is critical due to unpredictable tumor behavior and the possibility of a long period of dormancy. Complete resection of the solitary metachronous small bowel metastasis from RCC is indicated as a life-saving procedure and a curative treatment to cure metastasis-related complications. The present case report and review of the English-language literature emphasizes the rarity of this entity and recommends aggressive and tailored surgical treatment for these patients.

## Data availability statement

The original contributions presented in the study are included in the article/Supplementary Material. Further inquiries can be directed to the corresponding author.

## Ethics statement

The studies involving human participants were reviewed and approved by Ethical Committee on Biomedical Research, West China Hospital, Sichuan University. The patients/participants provided their written informed consent to participate in this study. Written informed consent was obtained from the individual for the publication of any potentially identifiable images or data included in this article.

## Author contributions

LY, WY, ZC, and PN conceptualized and designed the study. WY and ZC drafted the manuscript. TY and HZ were responsible for the literature search. QD, SQ, and JZ collected the clinical data. All authors contributed to the article and approved the submitted version.
